# COVID-19 Presenting as Acute Abdominal Pain: A Case Report

**DOI:** 10.7759/cureus.9659

**Published:** 2020-08-11

**Authors:** Nishan Purayil, Jaseem Sirajudeen, Naushad VA, Joe Mathew

**Affiliations:** 1 Internal Medicine, Hamad Medical Corporation, Doha, QAT

**Keywords:** covid-19, acute pancreatitis, acute abdomen

## Abstract

As the spread of severe acute respiratory syndrome coronavirus-2 (SARS-CoV-2) continues across the globe, more details about the disease manifestations and clinical course have been emerging. The main clinical presentation of the ongoing coronavirus disease-19 (COVID-19) pandemic is respiratory symptoms. Along with this, the involvement of the gastrointestinal system and associated symptoms have also been reported. Here we present a case of a 58-year-old patient who presented with acute abdominal pain and was diagnosed with acute pancreatitis. He did not have any respiratory symptoms, but had radiological evidence of lung involvement and was diagnosed to be positive for COVID-19.

## Introduction

The ongoing pandemic caused by severe acute respiratory syndrome coronavirus-2 (SARS-CoV-2) has so far caused more than 500,000 deaths worldwide [1]. The most common presentation of patients with coronavirus disease-19 (COVID-19) is respiratory symptoms like cough and shortness of breath, however, gastrointestinal symptoms like abdominal pain, diarrhea, and vomiting are also reported. The detection of viral ribonucleic acid (RNA), in fecal specimens in patients with negative respiratory swab results, suggests a possibility of feco-oral transmission [2].

Acute pancreatitis is one of the causes of acute abdominal pain in patients presenting to the emergency department. The development of acute pancreatitis is multifactorial and at times, the exact etiology cannot be identified. The most common cause is gallstones and alcohol abuse, but viral-induced acute pancreatitis has also been described [3]. This case report describes a patient with no precipitating factors presenting with abdominal pain to the emergency department; the patient was found to have pancreatitis and was screened positive for COVID-19.

## Case presentation

**Figure 1:**
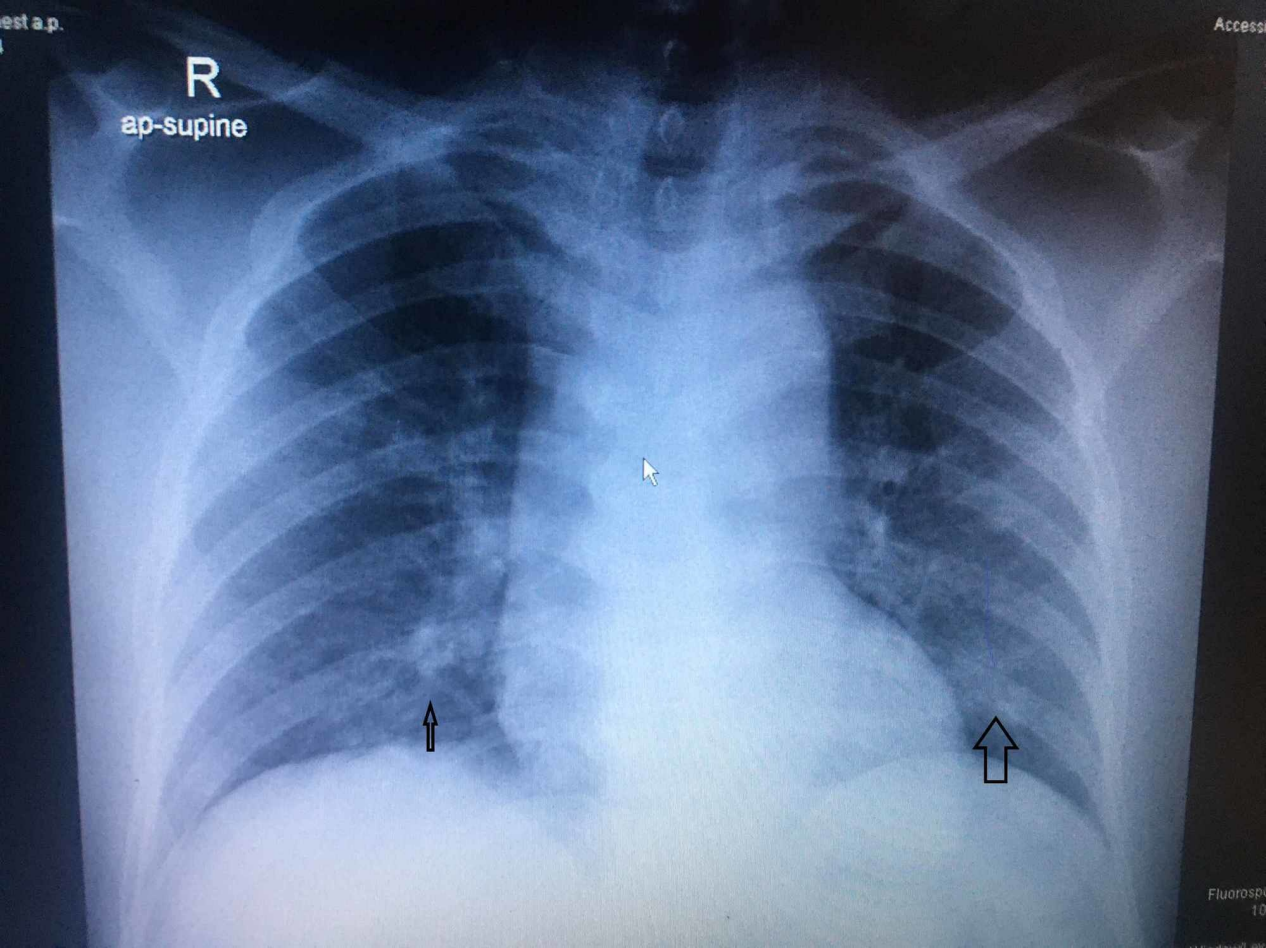
Chest X-ray showing basal infiltrates

A 58-year-old male patient presented with fever and vomiting for three days. This was associated with epigastric pain. No diarrhea. No respiratory symptoms. No urinary complaints. He was not on any regular medication. The examination was unremarkable except for mild epigastric tenderness. He was evaluated; his blood counts and liver function tests were normal (Table 1). His lipase was >600 U/L and amylase was 249 U/L. The chest X-ray revealed bilateral infiltrates (Figure 1). In view of the chest X-ray detecting COVID-19, polymerase chain reaction (PCR) screening was done which turned out to be positive. The patient was started on azithromycin and hydroxychloroquine (as per the COVID treatment protocol) along with supportive measures. The ultrasound scan of the abdomen was unremarkable (pancreas not visualized). His lipid panel was within normal limits. On further inquiry, he denied the consumption of alcohol or any new medications. With supportive treatment, he improved clinically. Abdominal pain improved and the patient was tolerating oral feeds. Later, he developed diarrhea and vomiting which was manged with anti emetics and intravenous fluids. He improved clinically and was transferred to a quarantine facility.

**Table 1:** Lab results AST: aspartate aminotransferase; ALT: alanine aminotransferase; ALP: alkaline phosphatase; CRP: C-reactive protein.

Test	result	Normal Range
Bilirubin	7	0-21 micromol /L
AST	25	0- 41 U/L
ALT	48	0- 41 U/L
ALP	44	40 -129 U/L
Lipase	>600	13-60 U/L
Amylase	249	13-60 U/L
CRP	29	0-5

## Discussion

The diagnostic criteria for acute pancreatitis requires the presence of two out of the following three criteria: 1) abdominal pain; 2) elevated amylase or lipase (more than three times the upper limit); 3) characteristic finding on imaging studies^ ^[4]. Studies have reported viral infection as one of the causes of acute pancreatitis. Infections like mumps, measles, coxsackie, Epstein-Barr virus, and hepatitis-A virus are commonly associated with pancreatitis [5]. It is reported that coronavirus infects human and animal species producing a myriad of symptoms [6,7]. A report from China described the presence of acute pancreatic injury in about 17% of cases diagnosed to have COVID-19. The high affinity of SARS-CoV-2 to the angiotensin-converting enzyme-2 (ACE-2) is explained in the pathogenesis of COVID-19 [8]. The presence of ACE-2 receptors in pancreatic cells is being attributed to pancreatitis in patients with COVID-19. However, in a series of 71 cases, McNabb-Baltar et al. reported patients having elevated serum amylase levels with no other features of pancreatitis [9].

Acute pancreatitis in patients with COVID-19 could be due to the direct cytotropic effect of the virus or due to the sequel of the immune response of the body. Szatmary et al. observed that the clinical course of pancreatitis in patients with COVID-19 is much more benign than expected; they also postulated pancreatico-duodenal inflammation with steatosis as a possible etiology [10]. In another study from Northern California, Gubatan et al. explored the possibility of patients with a past history of pancreatitis being more susceptible to COVID-19, however, the results were inconclusive [11].

The patient developed symptoms like diarrhea and vomiting during the stay in hospital which can be due to the COVID-19 infection or can be due to the medications. 

## Conclusions

A wide spectrum of presentation and system involvement makes screening for COVID-19 important in patients presenting to the emergency department. No obvious cause for pancreatitis could be identified in this patient. Early identification and appropriate treatment is vital for good prognosis. Atypical presentation and multisystem involvement is a real challenge for frontline clinicians managing the pandemic.
